# Supplementary material for: Modeling glioblastoma heterogeneity as a dynamic network of cell states
Source: Mol Syst Biol. 2021 Sep 16;17(9):e10105. doi: 10.15252/msb.202010105 (PMC8444284; doi:10.15252/msb.202010105)
Supplement: Supplementary file 5 — Source Data for Figure 3 [file MSB-17-e10105-s001.zip › Figure3A_sourcedata/GSEA_3065/hallmarks_state4.GseaPreranked.1623416296068/HALLMARK_SPERMATOGENESIS.html]

Details for gene set HALLMARK\_SPERMATOGENESIS[GSEA]

|  || Dataset | state4 |
| Phenotype | NoPhenotypeAvailable |
| Upregulated in class | na\_neg |
| GeneSet | HALLMARK\_SPERMATOGENESIS |
| Enrichment Score (ES) | -0.6649849 |
| Normalized Enrichment Score (NES) | -1.9882807 |
| Nominal p-value | 0.0 |
| FDR q-value | 0.0 |
| FWER p-Value | 0.0 |
Table: GSEA Results Summary

  

Fig 1: Enrichment plot: HALLMARK\_SPERMATOGENESIS      
 Profile of the Running ES Score & Positions of GeneSet Members on the Rank Ordered List

  

| PROBE | GENE SYMBOL | GENE\_TITLE | RANK IN GENE LIST | RANK METRIC SCORE | RUNNING ES | CORE ENRICHMENT || 1 | PCSK1N |  |  | 172 | 0.212 | 0.0096 | No |
| 2 | CNIH2 |  |  | 368 | 0.160 | 0.0064 | No |
| 3 | PEBP1 |  |  | 449 | 0.144 | 0.0187 | No |
| 4 | GSTM3 |  |  | 645 | 0.121 | 0.0086 | No |
| 5 | TALDO1 |  |  | 830 | 0.101 | -0.0032 | No |
| 6 | MLF1 |  |  | 1635 | 0.058 | -0.1222 | No |
| 7 | RAD17 |  |  | 3304 | 0.018 | -0.3867 | No |
| 8 | CSNK2A2 |  |  | 3327 | 0.017 | -0.3872 | No |
| 9 | NPHP1 |  |  | 3421 | 0.015 | -0.3995 | No |
| 10 | PRKAR2A |  |  | 3609 | 0.009 | -0.4280 | No |
| 11 | IFT88 |  |  | 3800 | 0.001 | -0.4584 | No |
| 12 | SPATA6 |  |  | 4039 | -0.012 | -0.4946 | No |
| 13 | BRAF |  |  | 4397 | -0.036 | -0.5456 | No |
| 14 | ARL4A |  |  | 4864 | -0.073 | -0.6077 | No |
| 15 | VDAC3 |  |  | 4979 | -0.081 | -0.6118 | No |
| 16 | PARP2 |  |  | 5119 | -0.093 | -0.6178 | No |
| 17 | JAM3 |  |  | 5414 | -0.120 | -0.6440 | Yes |
| 18 | NEK2 |  |  | 5505 | -0.132 | -0.6353 | Yes |
| 19 | PSMG1 |  |  | 5518 | -0.133 | -0.6138 | Yes |
| 20 | NCAPH |  |  | 5595 | -0.144 | -0.6009 | Yes |
| 21 | IL13RA2 |  |  | 5658 | -0.153 | -0.5841 | Yes |
| 22 | TTK |  |  | 5684 | -0.157 | -0.5605 | Yes |
| 23 | PIAS2 |  |  | 5753 | -0.173 | -0.5411 | Yes |
| 24 | TOPBP1 |  |  | 5758 | -0.174 | -0.5113 | Yes |
| 25 | SLC12A2 |  |  | 5790 | -0.181 | -0.4846 | Yes |
| 26 | KIF2C |  |  | 5996 | -0.237 | -0.4760 | Yes |
| 27 | AURKA |  |  | 6026 | -0.248 | -0.4372 | Yes |
| 28 | BUB1 |  |  | 6045 | -0.266 | -0.3934 | Yes |
| 29 | CCNB2 |  |  | 6140 | -0.329 | -0.3509 | Yes |
| 30 | EZH2 |  |  | 6159 | -0.348 | -0.2928 | Yes |
| 31 | CDKN3 |  |  | 6160 | -0.349 | -0.2316 | Yes |
| 32 | DBF4 |  |  | 6180 | -0.387 | -0.1668 | Yes |
| 33 | RFC4 |  |  | 6185 | -0.399 | -0.0974 | Yes |
| 34 | CDK1 |  |  | 6246 | -0.627 | 0.0027 | Yes |
Table: GSEA details [plain text format]

  

Fig 2: HALLMARK\_SPERMATOGENESIS: Random ES distribution      
 Gene set null distribution of ES for **HALLMARK\_SPERMATOGENESIS**
